# Using Claims Data to Examine Patients Using Practice-Based Internet Communication: Is There a Clinical Digital Divide?

**DOI:** 10.2196/jmir.6.1.e1

**Published:** 2004-01-09

**Authors:** Susan H Swartz, Timothy M Cowan, Ida A Batista

**Affiliations:** ^1^Center for Outcomes Research and EvaluationMaine Medical CenterPortland MEUSA

**Keywords:** Internet, ambulatory care, primary health care, claims analysis, outpatients, ICD codes, ICD-9, chronic disease, communication, patient education

## Abstract

**Background:**

Practice-based Internet communication allows patients to obtain health information, ask questions, and submit requests through a personalized Web site. While such online tools also bring great promise for educating patients with the goal of fostering behavior change, it is important to examine how individuals currently using such services differ from those who do not.

**Objective:**

The study used administrative information to characterize a population of patients communicating with a medical practice through the Internet during the end of 1999 and through 2000.

**Methods:**

Patient claims data generated during clinical encounters from January 1999 through May 2000 were examined to measure the relationship between patient demographics, frequency of visits, specific acute diagnoses, and specific chronic diagnoses and the use of online communication with the practice.

**Results:**

Ten percent of patients, and 13.2% of patients 18 years or older, used the practice Web site. There were differences in use of the practice Web site by age and insurance status, but not by gender. Use of the practice Web site was similar or higher among patients having a diagnosis for a variety of acute and chronic conditions compared to those not having such a diagnosis. Patients with more clinic visits were more likely to use the Web-based service.

**Conclusions:**

Patients using practice-based Internet communication and having significant health risks can be identified through the use of administrative data, presenting an opportunity to test online educational efforts to improve health.

## Introduction

Using Internet technology to improve health outcomes is a reasonable but still-unrealized goal. An increasing number of Internet services provide support for individuals with certain diagnoses, and there is rapid development of new computer-based programs targeting chronic conditions such as diabetes [1] and smoking [2]. Health care systems should perceive Internet communications as an important component in providing comprehensive, quality services with the potential to enhance interactions between patients and providers [3,4].

Practice-based Internet communication offers patients a secure method to query providers, obtain health information, and access personal clinical information [5]. More importantly, online messaging with practices provides opportunities to educate patients and, ideally, to improve their health [6]. Although use of Internet practice-patient communication remains low, studies reveal that a majority of individuals favor e-mailing providers about health questions [7] and are not appreciably concerned about privacy issues [8]. Patients currently communicating online with providers report high levels of satisfaction with these services and find them easy to use [9].

As the United States online population (58% of adults in 2002) rises, there is data to suggest that Internet patterns are changing and more dynamic [10]. Evidence consistently shows that those who are currently online seek health-related information to gain education about a specific disease or medical problem [11]. A recent Pew Internet & American Life Project survey found that Internet users who report having a chronic illness or disability search for information about medical problems or treatments more frequently, and are more likely to e-mail providers or loved ones about a health issue [12]. Houston and Allison, in a similar study, found that those rating their health as only fair or poor are significantly more likely to e-mail their doctor or use an online group than those rating themselves healthier [13]. These data lead to new questions about Internet users: do those diagnosed with new medical conditions subsequently become more avid eHealth seekers? Or are eHealth seekers worried about their health and have few medical problems and chronic conditions?

We examined the clinical characteristics of patients using a newly-established practice-based Internet communication tool. We hypothesized that patients having a wide variety of medical conditions were as likely to communicate online with their providers as those without such conditions. Our particular interest was to examine, using administrative claims data, the relationship between Internet use and having a diagnosis for a chronic disease.

## Methods

The study site was 1 of 4 clinics at Martin's Point Health Care [14], a nonprofit organization in northern New England, with 30 primary care physicians caring for approximately 50000 patients having 135000 visits annually. The study population included all patients having a claim at the study clinic site between January 1, 1999 and May 30, 2000. The Institutional Review Board at Maine Medical Center approved the study.

Beginning in late summer of 1999, the study clinic offered an online practice-patient interface. This secure online information system was designed to enhance patient education and communication, enabling patients to schedule an appointment, request medication refills, or ask nonurgent medical questions [15]. The Internet program was developed and maintained by MediVation. MediVation was purchased by McKesson in late 2000; shortly thereafter, the company stopped all support of the Internet program.

All patients having a primary care provider at the study clinic were mailed information about the Web-based communication program and invited to register. Patients were also informed of the service during clinic visits. To register, patients were required to log on to the practice Web site and enter personal information and a medical record number previously provided to them. A home page was created for each individual, personalized with provider and patient-specific information. The software automatically populated registered users' pages with appointment data and limited diagnosis data generated from the practice administrative and billing systems. Each patient page also contained links to health information related to specific conditions or clinical encounters. The clinic was not using an electronic medical record. For patients younger than 18 years, there was no information available on whether the patient or the parent had registered and was using the service.

The investigators were provided with administrative data on all patients having a claim at the study clinic between January 1, 1999 and May 30, 2000 (predating the start of the Internet service). The patient data included a unique identifier, age, gender, and insurance information. Administrative claims included Current Procedural Terminology (CPT) codes for evaluation and management (E & M) and laboratory, imaging, and other procedures performed during outpatient visits. Claims data also included diagnostic codes linked to the visits or procedures, based on the International Classification of Diseases, Ninth Revision (ICD-9). The data file identified all patients registering for the practice-based Internet communication service from August 1, 1999 through November 1, 2000. Both files were linked, allowing identification of patients using the practice Web site (referred to here as practice Web site Users or as Users) or not using the practice Web site (referred to here as practice Web site Nonusers or as Nonusers).

To examine the frequency of specific diagnoses among Users and Nonusers, we utilized Clinical Classifications Software (CCS), a diagnosis and procedure categorization scheme that classifies discrete sets of acute and chronic conditions [16]. The Clinical Classifications Software collapses the large number of ICD-9 codes into a smaller number of clinically-meaningful categories. To identify patients with a specific condition, all primary and secondary ICD-9 codes linked to outpatient evaluation and management claims were examined. Four diagnostic categories were examined in patients younger than 18 years, and 13 categories were studied in patients 18 years or older. These diagnoses were selected because they are either commonly seen in primary care or are conditions where education and patient-provider communication may play an important role in long-term outcomes. Respiratory infections included pneumonia, influenza, tonsillitis, bronchitis, and other upper-respiratory infections. The coronary artery disease group included claims for acute myocardial infarction, coronary atherosclerosis, and congestive heart failure.

Frequencies and chi-square tests of independence were used to examine differences in the patients using the practice Web site by demographic subgroup. Patient age was calculated as age at the beginning of the study period (January 1, 1999). The total number of outpatient visits (evaluation and management Current Procedural Terminology codes 99201-99215, 99381-99404) and the number of preventive health or counseling visits (Current Procedural Terminology codes 99381-99404 only) during the study interval were examined. Chi-square tests of independence were used to compare the frequency of visits and prevalence of selected diagnoses among Users and Nonusers.

## Results

There were 9781 unique patients identified as having at least 1 claim during the study interval. The clinic population was 55.1% female, with 45.1% younger than 18 years, 12.4% aged 18 to 29, 23.9% aged 30 to 49, and 18.6% aged 50 or older. Among patients with a claim, 982 or 10.0% registered online to use the practice tool from August 1999 through 2000. Of patients 18 years or older, 13.2% were Users.

Table 1 shows the demographic characteristics of Users compared with all patients having any claim at the clinic. While patients within each age cohort registered, a significantly higher proportion of those aged 50 to 69 were Users (16.5%), compared to those younger than 18 years (6.4%), aged 18 to 39 (10.9%), and aged 70 or older (5.9%). Similar proportions of male and female patients were Users. A higher proportion of patients insured by the Uniformed Services Family Health Plan (an insurance plan for active and retired military enrollees) were Users (15.5%), compared to other insured patients. Both Medicaid and Medicare beneficiaries seen at the clinic were less likely to use the Internet service than other insured patients. (Medicaid is a program for those unable to afford regular medical service; it is financed by the state and federal governments. Medicare is a government program of medical care especially for the aged.)

**Table 1:** Proportion of patients using the practice Web site, by demographic characteristics

**Patient Demographic Characteristic**		**All Patients With Demographic Characteristic**	**Users** **of Practice Web Site**		Χ ^2^(df)*	P*
		**N**	**N**	**%**		
Patients with any claim, January 1, 1999 to May 30, 2000		9781	982	10.0		
					
Age					178.4 (6)	< .001
	Younger than 18 years	4409	283	6.4		
	18-29 years	1210	113	9.3		
	30-39 years	1229	153	12.5		
	40-49 years	1109	166	15.0		
	50-59 years	847	143	16.9		
	60-69 years	655	105	16.0		
	70 years and older	322	19	5.9		
					
Gender					0.6 (1)	.43
	Female	5392	553	10.3		
	Male	4389	429	9.8		
					
Insurance					225.5 (5)	< .001
	US Family Health Plan†	3705	573	15.5		
	Commercial fee for service (FFS)/ health maintenance organization (HMO)	3844	320	8.3		
	Medicaid‡	605	23	3.8		
	Medicare§	348	13	3.7		
	Other	121	9	7.4		
	None or Unknown	1158	44	3.8		

^*^ Tests compared the proportions of patients using the practice Web site within subgroups by patient age, gender, and insurance status.

^†^ US Family Health Plan is an insurance plan for active and retired military enrollees.

^‡^ Medicaid is a program for those unable to afford regular medical service; it is financed by the state and federal governments.

^§^ Medicare is a government program of medical care especially for the aged.

The pattern of outpatient utilization among Users and Nonusers is shown in Table 2. Patients having more clinic visits during the study interval were significantly more likely to be Users. A greater proportion of Users had 4 or more visits during the study interval (55.6%), compared to Nonusers (37.1%). Users were also more likely to have clinical encounters coded as a preventive visit. The mean number of visits to the clinic during the 17-month study interval was 4.9 for Users and 3.6 for Nonusers.

**Table 2:** Outpatient visits among Nonusers and Users of the practice Web site

**Number of Visits**	**Nonusers of Practice Web Site** **(N = 8799)**		**Users of Practice Web Site** **(N = 982)**		Χ ^2^(df)*	P*
	N	%	N	%		
Outpatient visits†					170.5 (5)	< .001
	0	205	2.3	16	1.6		
	1	2332	26.5	112	11.4		
	2-3	2997	34.1	308	31.4		
	4-6	2026	23.0	310	31.6		
	7-9	724	8.2	131	13.3		
	10-31	515	5.9	105	10.7		
Preventive visits‡					114.2 (2)	< .001
	0	3979	45.2	275	28.0		
	1	4120	46.8	579	59.0		
	2 or more	700	8.0	128	13.0		

^*^ Tests compared the distribution of visits by Users to visits by Nonusers.

^†^ Evaluation and management claims with Current Procedural Terminology code 99201-99215 (outpatient office visit) or 99381-99404 (preventive medical services or counseling).

^‡^ Evaluation and management claims with Current Procedural Terminology code 99381-99404 only.

Table 3 and Table 4 examine the frequency of claims for several conditions seen during ambulatory visits by Users and Nonusers. Among patients younger than 18 years, there was no significant relationship between being a User and having a claim for either an eye or ear infection. In patients younger than 18 years, there was a direct association with having a diagnosis for respiratory infection or asthma and being a Use r. Of patients younger than 18 years (or their parents) who were Users, 16.1% had at least 1 claim for asthma, compared to 9.4% of those who were not using the service. Among all patients under age 18 with a diagnosis of asthma, 10.6% were Users, compared to 6.0% of those not having this diagnosis. In patients 18 years or older, those having a claim for a respiratory, skin, or urinary-tract infection were equally likely to be Users. Patients 18 years or older having clinic visits for a back problem, headache, or depressive disorder, however, were significantly more likely to be Users.

**Table 3:** Frequency of ICD-9* claims for specific diagnoses in Users and Nonusers of the practice Web site

**Patient**	**Nonusers of Practice Web Site** **(N = 4058) %**	**Users of Practice Web Site** **(N = 279) %**	**Χ ^2^(df)†**	***P†***
Patients younger than 18 years:				
	Eye infection	10.3	9.7	0.1 (1)	.75
	Otitis media	27.0	31.2	2.4 (1)	.13
	Respiratory infections‡	51.6	63.1	13.7 (1)	< .001
	Asthma	9.4	16.1	13.4 (1)	< .001
Patients 18 years or older:				
	Respiratory infections‡	33.4	34.2	0.7 (1)	.17
	Urinary tract infections	4.8	6.4	3.1 (1)	.08
	Skin infections	7.1	8.3	1.3 (1)	.26
	Back problems	12.3	16.7	10.4 (1)	.001
	Headaches, including migraines	5.8	9.2	11.2 (1)	< .001
	Depressive disorder§	5.7	8.4	7.7 (1)	.006

^*^ International Classification of Diseases, Ninth Revision.

^†^ Tests compared the proportion of Users with ICD-9 code(s) to that of Nonusers.

^‡^ Includes claims for pneumonia, influenza, tonsillitis, bronchitis, other respiratory infections.

^§^ Includes claim for "depressive disorder not elsewhere classified" (ICD-9 code = 311); it does not include major depression, manic-depressive or bipolar disorder.

The frequency of having a diagnosis claim for selected chronic conditions is shown in Table 4. Use of the practice Web site did not vary significantly among patients having a claim for a chronic diagnosis, except for patients having a claim for lipid disorder. The most prevalent diagnosis, hypertension, was not related to use of the practice Web site. Claims for tobacco use, alcohol abuse, or other substance abuse—ICD-9 codes that providers variably use during clinical encounters—are seen equally across both cohorts (Nonusers and Users). Having any 1 of the 7 chronic conditions was significantly related to use of the practice Web site; however, this was mostly attributable to having a claim for lipid disorder. Among all patients, the diagnostic category with the highest level of use of the practice Web site was patients 18 years or older having a lipid disorder, at 20%.

**Table 4:** Frequency of ICD-9* claims for chronic diagnoses in Users and Nonusers of the practice Web site, for patients 18 years or older

**Chronic Condition**	**Nonusers** **of Practice Web Site** **(N = 4536)** **%**	**Users** **of Practice Web Site** **(N = 687)** **%**	**Χ ^2^(df)†**	***P†***
Hypertension	15.2	15.7	0.1 (1)	.73
Coronary disease‡	2.9	2.6	0.2 (1)	.69
COPD	2.4	2.0	0.3 (1)	.56
Diabetes	5.5	5.7	< 0.1 (1)	.88
Lipid disorder	8.9	14.7	23.2 (1)	< .001
Alcohol or substance abuse	7.2	7.3	< 0.1 (1)	.95
Tobacco user disorder	7.1	6.8	0.1 (1)	.77

^*^ International Classification of Diseases, Ninth Revision.

^†^ Tests compared the proportion of Users with ICD-9 code(s) to that of Nonusers.

^‡^ Includes acute myocardial infarction, coronary atherosclerosis, or congestive heart failure.

## Discussion

This study offers a real-world glimpse into the clinical footprints of a patient population communicating online with a practice. Using administrative claims data, we examined the diagnoses and clinic visits of 2 cohorts—those using and not using the practice Web site to obtain health information and make requests online. The Users, 10% of the clinic population, appeared demographically and clinically diverse. Of note, while patients aged 50 to 69 comprised 15% of the clinic population, they comprised 25% of the User group.

Overall, our findings suggest that patients with higher outpatient utilization have a stronger preference for online practice-based communication, but are not just the "worried well." Use of the practice Web site was higher in patients with pediatric asthma (or their parents), as well as in patients 18 years or older with visits for back pain, depression, lipid disorder, and multiple chronic conditions. Indeed, 1 out of 3 Users 18 years or older had been given a diagnosis for at least 1 chronic condition during the relatively-short study interval. It is likely that several factors affected the degree to which patient subgroups might use the practice Web site. Patients needing ongoing prescriptions, such as those with asthma, lipid disorder, or depression, may have been drawn to requesting refills online. Additionally, those having more clinic visits may have received more encouragement by their doctor or nurse to submit questions online following a clinical encounter.

Several study limitations should be mentioned. First, 90% of patients were not Users. However, the study examined online patient-practice communication in 1999 and 2000, representing a very early "era" of such services. Second, we did not have education or income information, and examined only claims generated from clinical encounters at the study clinic. We found less Internet use among Medicaid recipients, suggesting that lower income was an important factor in online practice communication. Third, as Users had more visits, it is possible that patient registration for the service was related to recruitment efforts. Indeed, the group with the highest representation was the Uniformed Services Family Health Plan enrollees; this group received additional outreach at the clinic site. In spite of this, finding similar frequencies of chronic diagnoses, including alcohol and tobacco use, suggests that exposure to marketing or clinic visits may have been less of a factor in engaging patients to use the practice Web site. Finally, we did not know how patients used the practice Web site after online registration; this analysis was planned, but was unable to be performed after the sale of the Internet company.

While research shows that Internet access tends to be higher in younger, healthier patients, eHealth seekers most often use the Internet to find information about a disease or medication, or to help change behavior [17]. Our study of a single clinic population shows that Users of a practice-based Web site are also older patients and those with chronic disorders. The results support other studies showing that individuals with chronic conditions are active eHealth seekers. In a large national survey examining the extent of eHealth use, Baker et al found that respondents self-reporting worse health status were more likely to use the Internet for health and health care [7]. Furthermore, among those reporting 1 or more chronic conditions, 58% perceived eHealth to improve understanding of the condition, 27% felt self-management improved, and only 7% thought that eHealth led them to seek care from another provider. With sicker patients seemingly showing higher interest in "pushing" eHealth, future efforts should focus on "pulling" these cohorts to be more informed and active in self-care and chronic disease management [18- 20]. Interactive technology can play an important role in health-status assessment and tailored feedback [21], and potentially increase patient self-efficacy and empowerment [22].

Future studies on practice-based Internet communication should be more comprehensive and examine patterns of online activity, as well as include socioeconomic demographics. Researchers and innovators are on an early part of the learning curve in using online methods to improve preventive care, manage chronic disease, and modify behavior. Such studies should also evaluate all patient-level claims, including inpatient and pharmacy utilization. Patients having a chronic disorder, as well as those identified with other conditions (eg, headache, back pain) might be appropriate candidates to test proactive patient education through Web-based communication. As cultural and health-system eHealth challenges continue to be addressed, perhaps finding that patients with chronic disorders are just as likely to communicate online with providers—ie, that there is no evidence of a clinical digital divide—will usher in real benefits for these patients with health risks.

## Abbreviations

ICD-9: International Classification of Diseases, Ninth Revision
